# Epigenetic modifications in *KDM* lysine demethylases associate with survival of early-stage NSCLC

**DOI:** 10.1186/s13148-018-0474-3

**Published:** 2018-04-02

**Authors:** Yongyue Wei, Junya Liang, Ruyang Zhang, Yichen Guo, Sipeng Shen, Li Su, Xihong Lin, Sebastian Moran, Åslaug Helland, Maria M. Bjaanæs, Anna Karlsson, Maria Planck, Manel Esteller, Thomas Fleischer, Johan Staaf, Yang Zhao, Feng Chen, David C. Christiani

**Affiliations:** 1 Department of Biostatistics, School of Public Health, Nanjing Medical University, Nanjing, 211166 China; 2 China International Cooperation Center (CICC) for Environment and Human Health, Nanjing Medical University, Nanjing, 211166 China; 3 Department of Environmental Health, Harvard T.H. Chan School of Public Health, Harvard University, Boston, MA 02115 USA; 4 Department of Biostatistics, Harvard T.H. Chan School of Public Health, Harvard University, Boston, MA 02115 USA; 5 Cancer Epigenetics and Biology Program (PEBC), Bellvitge Biomedical Research Institute (IDIBELL), 08908 L’Hospitalet, Barcelona, Catalonia Spain; 6 Department of Genetics, Institute for Cancer Research, Oslo University Hospital, Oslo, Norway; 7 Division of Oncology and Pathology, Department of Clinical Sciences Lund, Lund University, Medicon Village, SE 22381 Lund, Sweden; 8 Pulmonary and Critical Care Unit, Massachusetts General Hospital, Department of Medicine, Harvard Medical School, Boston, MA 02114 USA

**Keywords:** *KDM*, Lysine demethylase, DNA methylation, Lung cancer, Survival

## Abstract

**Background:**

*KDM* lysine demethylase family members are related to lung cancer clinical outcomes and are potential biomarkers for chemotherapeutics. However, little is known about epigenetic alterations in *KDM* genes and their roles in lung cancer survival.

**Methods:**

Tumor tissue samples of 1230 early-stage non-small cell lung cancer (NSCLC) patients were collected from the five independent cohorts. The 393 methylation sites in *KDM* genes were extracted from epigenome-wide datasets and analyzed by weighted random forest (Ranger) in discovery phase and validation dataset, respectively. The variable importance scores (VIS) for the sites in top 5% of both discovery and validation sets were carried forward for Cox regression to further evaluate the association with patient’s overall survival. TCGA transcriptomic data were used to evaluate the correlation with the corresponding DNA methylation.

**Results:**

DNA methylation at sites cg11637544 in *KDM2A* and cg26662347 in *KDM1A* were in the top 5% of VIS in both discovery phase and validation for squamous cell carcinomas (SCC), which were also significantly associated with SCC survival (*HR*_cg11637544_ = 1.32, 95%CI, 1.16–1.50, *P* = 1.1 × 10^−4^; *HR*_cg26662347_ = 1.88, 95%CI, 1.37–2.60, *P* = 3.7 × 10^−3^), and correlated with corresponding gene expression (cg11637544 for *KDM2A*, *P* = 1.3 × 10^−10^; cg26662347 for *KDM1A P* = 1.5 × 10^−5^). In addition, by using flexible criteria for Ranger analysis followed by survival classification tree analysis, we identified four clusters for adenocarcinomas and five clusters for squamous cell carcinomas which showed a considerable difference of clinical outcomes with statistical significance.

**Conclusions:**

These findings highlight the association between somatic DNA methylation in *KDM* genes and early-stage NSCLC patient survival, which may reveal potential epigenetic therapeutic targets.

**Electronic supplementary material:**

The online version of this article (10.1186/s13148-018-0474-3) contains supplementary material, which is available to authorized users.

## Background

Lung cancer, a highly invasive, rapidly metastasizing cancer, accounting for more than one million deaths each year, has been the leading cause of cancer deaths worldwide for decades [1]. Rapidly developing radiological techniques and strengthened consciousness of health screening result in an increased numbers of lung cancer patients diagnosed at early stage. Early-stage lung cancer patients are expected to have a better prognosis compared with those at late stage [2]. However, significant heterogeneity has been observed for clinical outcomes among the early-stage patients, which may have molecular mechanisms not well understood yet [3]. Epigenetic alterations—particularly methylation in target organ tissues—is a potential causation of this phenomenon [4].

Of particular interest, methylation changes at histone demethylase *KDM* gene family are broadly involved in cancer development [5, 6]. Because methylation is reversible, it provides a source of potential biomarkers and therapeutic targets in cancer [7, 8]. Most recent publications show a great potential of epigenetic modifiable agencies in cancer therapy [9, 10]. HumanN6-methyllysine residue demethylation is catalyzed by two distinct subfamilies of demethylases—the flavin-dependent *KDM1* subfamily and the JmjC-domain containing *KDM2-*8 subfamily, which regulate the chromatin state at specific loci, and impact gene expression, DNA repair, DNA replication, and genome stability [6]. Emerging evidence connects *KDMs* to cancers, including lung cancer [11–17]. DNA methylation is an important gene regulator that provides epigenetic therapeutic targets in cancer. However, to date, no studies have examined the role of DNA methylation in *KDM* demethylase genes and its relationship to clinical outcomes of lung cancer patients.

Therefore, we performed a large-scale association analysis between somatic DNA methylation in *KDM* gene family members and overall survival of non-small cell lung cancer (NSCLC) patients. The study was performed in a discovery set combining four independent Caucasian populations, followed by an independent replication in the data from The Cancer Genome Atlas (TCGA).

## Methods

### Lung cancer study populations

#### Harvard

The Harvard Lung Cancer Study cohort was described previously [18]. Briefly, all cases were recruited at Massachusetts General Hospital (MGH) since 1992 and were newly diagnosed, histologically confirmed primary NSCLC. Snap-frozen tumor samples were collected from NSCLC patients during curative surgery with complete resection. There were151 early-stage (TNM stage I, II) cases selected for this study which had complete survival information. Tumor DNA was extracted from 5-μm-thick histopathologic sections. Each specimen was evaluated by an MGH pathologist for amount (tumor cellularity > 70%) and quality of tumor cells and histologically classified using WHO criteria.

#### Spain

Study population was reported previously [19]. In brief, tumors were collected by surgical resection from patients who provided consent and under approval by the institutional review boards. The median clinical follow-up was 7.2 years.

#### Norway

As described previously [20], participants were patients with operable lung cancer tumors who were seen at Oslo University Hospital-Riks hospitalet, Norway, from 2006 to 2011. Only early-stage (stage I, II) patients were selected for the current study.

#### Sweden

Tumor tissue specimens were collected from patient’s early-stage lung cancer who underwent operation at the Skane University Hospital, Lund, Sweden [21].

#### TCGA

We used The Cancer Genome Atlas (TCGA) resources for validation, including 332 early-stage lung adenocarcinomas (AC) and 285 early-stage squamous cell carcinomas (SCC) which had survival information and common covariates. Level-1 HumanMethylation450 DNA methylation data (image data) of each patient were downloaded on October 01, 2015.

### Quality control process for DNA methylation data

DNA methylation was assessed using Illumina Infinium HumanMethylation450 BeadChips (Illumina Inc., San Diego, CA, USA). Raw image data were imported into Genome Studio Methylation ModuleV1.8 (Illumina Inc.) to calculate methylation signals and to perform normalization, background subtraction, and quality control. Unqualified probes were excluded, if they fit the following quality control (QC) criteria: (i) failed detection (*P* > 0.05) in ≥ 5% samples, (ii) coefficient of variance (CV) < 5%, (iii) methylated or unmethylated in all samples, (iv) common single nucleotide polymorphisms located in probe sequence or in 10-bp flanking regions, (v) or cross-reactive probes [22]. Samples with > 5% undetectable probes were excluded. Methylation signals were further processed for quantile normalization, type I and II correction, and batch effects adjustment using *ComBat* correction [23, 24]. Those QC and normalization processes were performed at each site separately using the same R code with identical settings. In addition, the site information was included as one of the covariates in the following models to control for potential site heterogeneity. Details of QC processes are described in Additional file 1: Figure S1.

### Gene expression data

In the TCGA cohort, all 332 AC and 285 SCC early-stage patients had complete mRNA sequencing data. TCGA mRNA sequencing data processing and quality control was done by the TCGA workgroup. Raw counts were normalized using RNA Sequencing by Expectation Maximization (RSEM). Level-3 (gene level) gene quantification data were downloaded from TCGA data portal and were further checked for quality. Expression of *KDM* genes was extracted and log_2_ transformed before analysis.

### Statistical analysis

Continuous variables were summarized as mean ± standard deviation (SD); categorical variables were described as *n* (%). Ranger, a weighted version of random forest for controlling for the potential confounders, was employed in discovery and validation set, respectively, to evaluate the importance of each individual methylation CpG site [25, 26]. A weight of 100% was given to each covariate to ensure a 100% chance to be selected into each tree. Variable importance score (VIS) for each methylation site was estimated and ranked in a descending order. CpG sites that were in top 5% in both discovery and validation set were identified as candidates and carried forward for traditional Cox regression.

The candidate methylation probes were further evaluated by Cox regression with adjustment for covariates including age, gender, smoking status, and stage. Results were described as hazard ratio (*HR*) and 95% confidence interval (95%CI) per 1% increment of methylation. Multiple testing corrections were performed using false discovery rate method (FDR; measured by FDR-*q* value) among results from discovery set. The sites with FDR-*q* ≤ 0.05 in discovery set were, in turn, validated in TCGA samples, with a statistical significance level of 0.05. Further, correlations between the DNA methylation of the validated sites and corresponding gene expression level was evaluated by linear regression using TCGA data. Only TCGA samples had both epigenome and transcriptome data, so the methylation-expression analysis was only performed in TCGA cohort.

In addition, flexible criteria were hired for further exploration: (1) CpG sites that were in top 10% in both discovery and validation set were identified as candidates, (2) candidates with FDR-*q* ≤ 0.1 in discovery set, and (3) *P* value ≤ 0.05 in validate set. Classification and regression tree (CART) was used to identify clusters with heterogeneous survival outcome. Kaplan-Meier method was used to illustrate the survival curves of different clusters.

All analyses were performed in R Version 3.2.4 (The R Foundation).

## Results

**Fig. 1:**
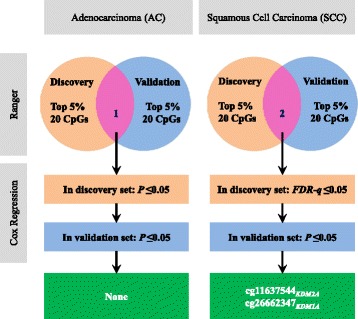
Analysis work flow. Adenocarcinoma and squamous cell carcinoma samples from Harvard, Spain, Norway, and Sweden cohorts were used for the discovery phase of analysis. Data from The Cancer Genome Atlas (TCGA) were used for validation. Ranger is a weighted version of random forest for controlling for the covariates including age, gender, smoking status, and histological stage. Variable importance score (VIS) was estimated for each CpG site and was ranked in descending order. CpG sites ranked in top 5% in both discovery and validation sets were selected for further evaluation by Cox regression. Multiple testing correction by false discovery rate (FDR) method was used if necessary

We analyzed 393 DNA methylation probes (Additional file 2: Table S1) in 17 *KDM* gene family members located on autosomal chromosomes (Additional file 3: Table S2). Analysis was performed on 1230 tumor DNA samples from early-stage NSCLC patients recruited from five lung cancer study cohorts. Demographics and clinical characteristics are described in Table 1. Figure 1 shows a flow chart for overall analysis. Samples from the Harvard, Spain, Norway, and Sweden studies were combined together for discovery.

**Table 1:** Demographic and clinic pathological descriptions for study populations

Variables	Discovery set					Validation set
	Harvard (*N* = 151)	Spain (*N* = 226)	Norway (*N* = 133)	Sweden (*N* = 103)	All (*N* = 613)	TCGA (*N* = 617)
Age (years), mean ± SD	67.67 ± 9.92	65.67 ± 10.58	65.52 ± 9.34	67.54 ± 9.99	66.44 ± 10.08	66.51 ± 9.47
Gender, *n* (%)						*
Female	67 (44.37)	105 (46.46)	71 (53.38)	54 (52.43)	297 (48.45)	255 (41.33)
Male	84 (55.63)	121 (53.54)	62 (46.62)	49 (47.57)	316 (51.55)	362 (58.67)
Smoking status, *n* (%)						*
Never	18 (11.92)	30 (13.57)	17 (12.78)	18 (17.48)	83 (13.65)	55 (9.18)
Former	81 (53.64)	120 (54.30)	74 (55.64)	54 (52.43)	329 (54.11)	376 (62.77)
Current	52 (34.44)	71 (32.13)	42 (31.58)	31 (30.10)	196 (32.24)	168 (28.05)
Unknown	0	5	0	0	5	18
TNM stage, *n* (%)						*
I	104 (68.87)	183 (80.97)	93 (69.92)	95 (92.23)	475 (77.49)	393 (63.70)
II	47 (31.13)	43 (19.03)	40 (30.08)	8 (7.77)	138 (22.51)	224 (36.30)
Histology, *n* (%)						*
Adenocarcinoma (AC)	96 (63.58)	183 (80.97)	133 (100.00)	80 (77.67)	492 (80.26)	332 (53.81)
Squamous cell carcinoma (SCC)	55 (36.42)	43 (19.03)	0 (0.00)	23 (22.33)	121 (19.74)	285 (46.19)
Chemotherapy, *n* (%)						*
No	142 (94.04)	177 (90.77)	102 (76.69)	67 (90.54)	488 (88.25)	194 (76.98)
Yes	9 (5.96)	18 (9.23)	31 (23.31)	7 (9.46)	65 (11.75)	58 (23.02)
Unknown	0	31	0	29	60	365
Radiotherapy, *n* (%)						
No	132 (87.42)	184 (94.36)	132 (99.25)	74 (100.00)	522 (94.39)	239 (94.84)
Yes	19 (12.58)	11 (5.64)	1 (0.75)	0 (0.00)	31 (5.61)	13 (5.16)
Unknown	0	31	0	29	60	365
Adjuvant therapy, *n* (%)						*
No	127 (84.11)	168 (86.15)	101 (75.94)	67 (90.54)	463 (83.73)	187 (74.21)
Yes	24 (15.89)	27 (13.85)	32 (24.06)	7 (9.46)	90 (16.27)	65 (25.79)
Unknown	0	31	0	29	60	365
Survival year, month						
Median (95%CI)	6.66 (5.41–7.87)	7.12 (5.06–9.63)	7.36 (6.77–7.95)^*^	7.39 (4.98–9.12)	7.39 (6.50–8.23)	4.54 (3.68–5.41)
Dead, *n* (%)	122 (80.79)	101 (44.69)	42 (31.58)	58 (31.58)	323 (52.69)	142 (23.01)

*Statistically significant difference (*P* ≤ 0.05) was observed between combined discovery set and validation set (TCGA)

**Fig. 2:**
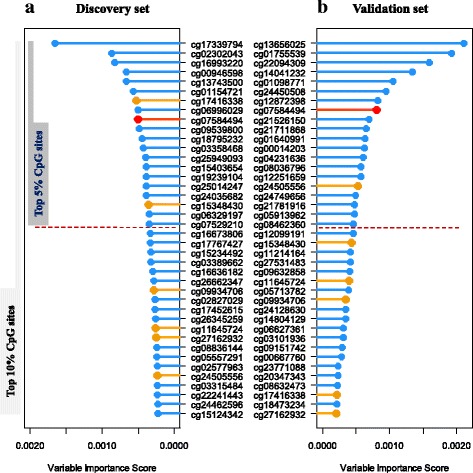
Ranger in discovery (**a**) or validation (**b**) in adenocarcinomas. Weighted random forest (Ranger, where confounders like age, gender, smoking status, and stage are adjusted with given 100% weight) was employed in discovery phase (**a**) and validation set (**b**) to evaluate the importance of variables. Ranger provides VIS (variable importance score) for each methylation sites. Variables that were in top 5% (red lollipop) or top 10% (yellow lollipop, a flexible criterion) in both discovery phase (**a**) and validation (**b**) would be carried forward

Among AC samples, only one common CpG site, cg07584494 in *KDM4B*, was identified of which the importance ranked in the top 5% both in discovery and validation sets (Fig. 2). This CpG was further evaluated by Cox regression, meanwhile, did not reach the stringent criteria of *P* value ≤ 0.05 in both discovery and validation sets.

**Fig. 3:**
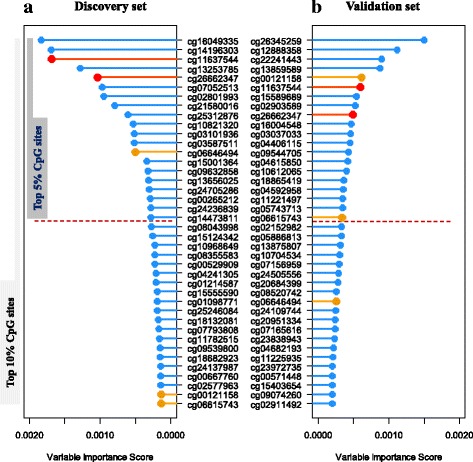
Ranger in discovery (**a**) or validation (**b**) in squamous cell carcinomas. Weighted random forest (Ranger, where confounders like age, gender, smoking status, and stage are adjusted with given 100% weight) was employed in discovery phase (**a**) and validation set (**b**) to evaluate the importance of variables. Ranger provides VIS (variable importance score) for each methylation sites. Variables that were in top 5% (red lollipop) or top 10% (yellow lollipop, a flexible criterion) in both discovery phase (**a**) and validation (**b**) would be carried forward

**Fig. 4:**
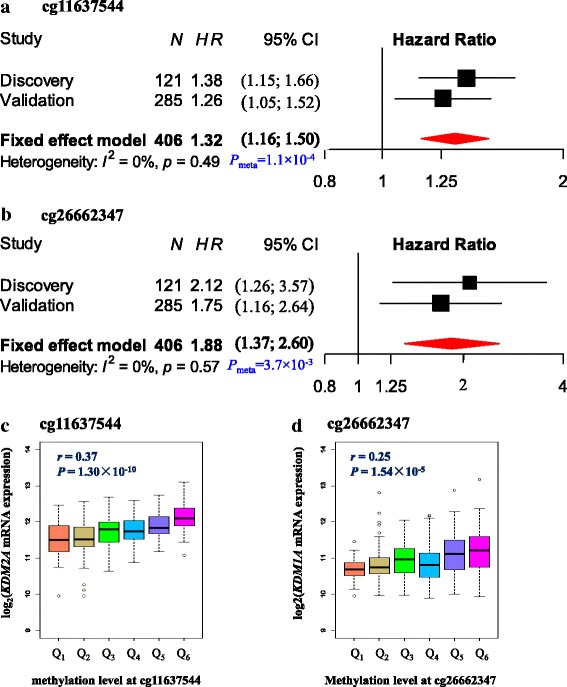
Association between DNA methylation at sites cg11637544 in *KDM2A* and cg26662347 in *KDM1A* with overall survival of squamous cell carcinomas and correlation between these two sites and their corresponding gene expression. Fixed-effects meta-analysis was used to combine the results from discovery and validation sets for squamous cell carcinomas (SCC) (**a** cg11637544_*KDM2A*_; **b** cg26662347_*KDM1A*_). *I*^2^and corresponding *P* value were used to evaluate heterogeneity across studies. DNA methylation level was categorized to six quantiles, and box plot for gene expression was drawn for each quantile. Pearson correlation was used to estimate the correlation coefficient (*r*) and the *P* value; gene expression was log2 transformed before analysis (**c** cg11637544_*KDM2A*_; **d** cg26662347_*KDM1A*_)

For SCC samples, the two common probes, cg11637544 within the first exon of *KDM2A* and cg26662347 at TSS200 region of *KDM1A*, were ranked in the top 5% in both discovery and validation sets (Fig. 3). These two CpG sites were further evaluated by Cox regression in discovery set and showed a significant association with SCC patients’ survival [cg11637544_*KDM2A*_, hazard ratio (*HR*) per 1% methylation increment = 1.38, 95% confidence interval (95%CI) 1.15–1.66, *P* = 0.0006, FDR*-q* = 0.0011; cg26662347_*KDM1A*_, *HR* = 2.12, 95%CI 1.26–3.57, *P* = 0.0045, FDR-*q* = 0.0090]. Both CpG sites were replicated with statistical significance in validation set from TCGA (*HR*_cg11637544_ = 1.26, 95%CI 1.05–1.52, *P* = 0.0136; *HR*_cg26662347_ = 1.75, 95%CI 1.16–2.64, *P* = 0.007). Meta-analysis combining the evidences from the discovery and validation sets further showed a stronger association between worse SCC patient’s survival and hyper-methylation at cg11637544_*KDM2A*_ (*HR* = 1.32, 95%CI, 1.16–1.50, *P* = 1.1 × 10^−4^; Fig. 4a) and at cg26662347_*KDM1A*_ (*HR* = 1.88, 95%CI, 1.37–2.60, *P* = 3.7 × 10^−3^; Fig. 4b). Furthermore, altered DNA methylation at sites cg11637544_*KDM2A*_ (*r* = 0.37, *P* = 1.3 × 10^−10^) (Fig. 4c) and cg26662347_*KDM1A*_ (*r* = 0.25, *P* = 1.5 × 10^−5^) (Fig. 4d) were positively correlated with the corresponding gene expression.

**Fig. 5:**
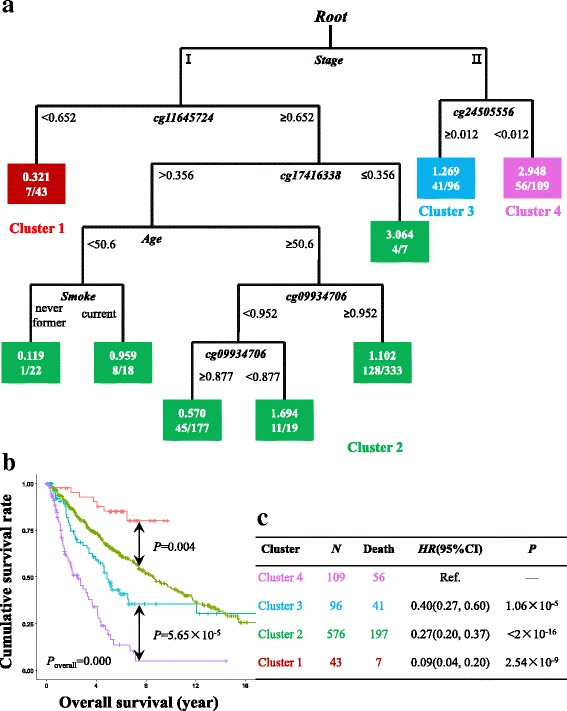
Survival classification tree for adenocarcinomas. Survival classification tree was built with seven CpG sites as well as covariates using the merged data of discovery and validation sets among adenocarcinoma cases (**a**), which identified five clusters with significantly different survival curves (**b**). Cox regression was used to compare the outcomes among clusters (cluster 4 as reference) and represented by hazard ratio (*HR*), 95% confidence interval (95%CI), and the *P* value (**c**)

**Fig. 6:**
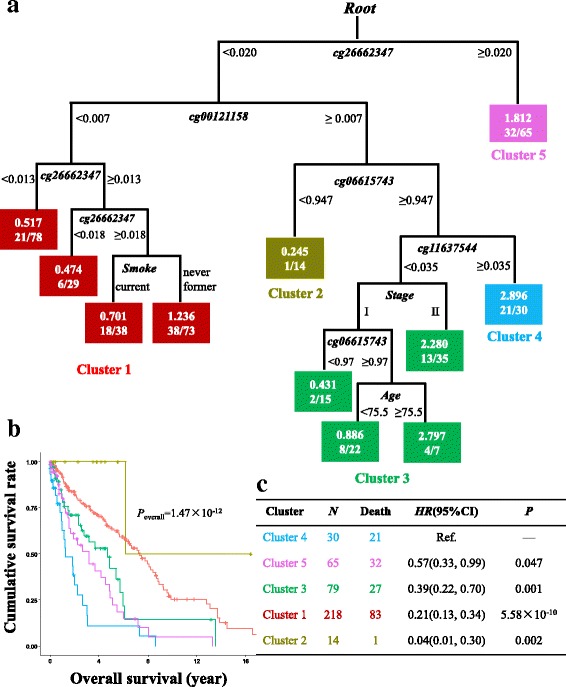
Survival classification tree for squamous cell carcinomas. Survival classification tree was built with five CpG sites as well as covariates using the merged data of discovery and validation sets among adenocarcinoma cases (**a**), which identified four clusters with significantly different survival curves (**b**). Cox regression was used to compare the outcomes among clusters (cluster 4 as reference) and represented by hazard ratio (*HR*), 95% confidence interval (95%CI), and the *P* value (**c**)

In addition to the stringent criteria, we also comprehensively explored the data using Ranger with relatively flexible criteria, followed by survival classification tree analysis which can consider both linear and non-linear patterns, and both individual and interaction effects of CpG sites simultaneously (Additional file 4: Figure S2). CpG sites of which the variable importance score ranked in the top 10% in both discovery and validation set were selected, including seven CpG sites for AC samples (Fig. 2) and five for SCC samples (Fig. 3). Among AC cases, seven CpG sites as well as covariates were used to build a survival classification tree using the merged data of discovery and validation sets (Fig. 5a). Four clusters were identified with significantly different survival curves (Fig. 5b) and outcome (Fig. 5c). Among these seven CpG sites, none showed a correlation with corresponding gene’s expression. Similarly, among SCC cases, five CpG sites, three of which were newly identified, and covariates were used to build the classification tree (Fig. 6a), which identified five clusters and statistically distinguished patient’s survival (Fig. 6b, c). Among the three newly identified CpG sites for SCC samples (Additional file 4: Figure S2), two of which were correlated with their corresponding gene expression with statistical significance (*r*_cg00121158_ = 0.24, *P*_cg00121158_ = 4.38 × 10^−5^; *r*_cg06615743_ = − 0.17, *P*_cg06615743_ = 0.004; Additional file 5: Figure S3A-B).

## Discussion

Several non-hypothesis-based epigenome-wide studies have analyzed lung cancer prognosis [19–21] and have identified several potential epigenetic biomarkers to help better understand the etiology of NSCLC. To the best of our knowledge, this is the first population-based study integrating five independent cohorts that suggested the relationship between tumor DNA methylation alterations at the *KDM* gene family members and NSCLC overall survival. The results expand our current understanding of the *KDM* gene family in lung cancer etiology.

DNA methylation surrounding the transcriptional start site (TSS) has been investigated extensively [27]; generally, hyper-methylation blocks transcription initiation and reduces gene expression [27, 28]. However, DNA methylation at cg11637544 in the first exon of *KDM2A* and at cg26662347 in the 200-kb TSS region of *KDM1A* elevates corresponding expression in tumor tissues. The function of gene body methylation remains unclear. However, functional elements could be in the gene body and could modulate expression through enhancers, transcription factor binding sites, and repetitive elements [29]. Gene body DNA methylation may maintain nucleosome stabilization in transcribed regions and increase transcriptional efficiency either by elongation or by splicing, which, in turn, leads to altered outcomes [30]. Although promoter-associated hyper-methylation mostly downregulates gene expression, a small part of methylation surrounding TSS region upregulates expression [31]. This phenomenon may be mediated by affecting the binding activity of upstream transcription factors [32], which warrants further well-designed functional studies.

As previously reported, overexpression of *KDM1A* and *KDM2A* in NSCLC cells increases cell proliferation and invasiveness and promotes cancer metastasis [12, 13]. Here, we provided further evidence that those associations appear to be generalizable to patient population. However, functional studies are needed to evaluate the mechanisms that underlie the associations between methylation alterations and survival and the mediating pathway affecting gene transcription. Nevertheless, no promising individual CpG site was identified for adenocarcinoma patients following the stringent criteria, which may due to underlying epigenetic heterogeneity between adenocarcinomas and squamous cell carcinomas [33, 34].

In addition, a comprehensive survival classification tree analysis were also performed to further explore the data by including more potentially associated CpG sites, which identified clusters with significantly different clinical outcome for both adenocarcinomas and squamous cell carcinomas. More *KDM* genes, including *KDM4B*, *KDM2B*, and *KDM4A* for adenocarcinomas and *KDM2B*, *KDM4C*, and *KDM4B* for squamous cell carcinomas, appear to be associated with patients’ survival. So far, *KDM2B* has been involved in mechanisms, independently or interactively with microRNAs, for cancers of the blood [35], pancreas [36], breast [37], or stomach [38]. However, the direct impact of *KDM2B* DNA methylation on SCC survival is not well understood and therefore is an area that needs further exploration. *KDM4* histone demethylases is emerging as key regulatory modifiers to histone trimethylated residues that have an important role in cancer development and as a potential therapeutic targets [39]. *KDM4A* is related to mTOR inhibitor sensitivity in SCC patients and impacts copy gains of drug-resistant regions in the genome [17, 40]. *KDM4B* encodes a DNA damage response protein that confers a survival advantage following γ-irradiation [41]. *KDM4C* inhibition by curcuminoids is an adjuvant therapy that can benefit colon cancer patients [42]. However, methylation signals in *KDM4* family members are not individually associated with NSCLC survival, which indicates that there may have interactions of *KDM4* and the other elements.

A major strength of this study is that we used weighted random forest (Ranger) to filter DNA methylation signals. Random forest (RF) is powerful in handling high-dimensional genetic data, but false positive or spurious association may occur if confounding factors are not corrected [43]. In Ranger, there is a parameter from 0 to 1 that represents the probability of variables selected for splitting the tree [26]. If the weight of covariate is given a value of 1, this covariate would be involved in each tree with 100% chance, and thus be controlled. In addition, survival classification tree, a nonparametric and decision-tree-based data mining approach, was used which can improve statistical power and indicate potential interactions between the CpG sites in this study [44, 45]. However, further studies need to be done to validate authenticity and how they interact, which is another area that needs further exploration.

We acknowledge some limitations in our study. First, the positive association between methylation and corresponding gene expression lacks biological evidence. The association should be interpreted with caution, and thus warrants further functional experiments. Second, the censored rate of TCGA cohort is relatively high. Early-stage NSCLC patients could be followed longer to obtain more precise estimates in future. In addition, clinical therapy information after surgery is under informative and not included in this study. Finally, our study did not include other races rather than Caucasian. The findings of this study should be interpreted with caution among other populations. Further studies are needed to investigate their possible differences among multiple ethnicities.

## Conclusions

In conclusion, this study highlights the role of somatic epigenetic alterations of *KDM* gene family members on NSCLC overall survival. The findings indicate potential dynamic and reversible therapeutic targets for lung cancer patients and may suggest a high-risk early-stage NSCLC population for adjuvant therapies.

## Additional files

Additional file 1: **Figure S1.** Quality control processes for DNA methylation chip data. Quality control measures and exclusion criteria as applied to Harvard, Spain, Norway, Sweden, and The Cancer Genome Atlas (TCGA) sample cohorts. (PDF 642 kb)

Additional file 2: **Table S1.** Annotation of CpG sites in KDM gene family. (PDF 677 kb)

Additional file 3: **Table S2.** Distributions of CpG sites in KDM genes. (PDF 153 kb)

Additional file 4: **Figure S2.** Analysis work flow. Adenocarcinoma and squamous cell carcinoma samples from Harvard, Spain, Norway, and Sweden cohorts were used for the discovery phase of analysis. Data from The Cancer Genome Atlas (TCGA) were used for Validation. Ranger is a weighted version of random forest for controlling for the covariates including age, gender, smoking status, and histological stage. Variable importance score (VIS) was estimated for each CpG site and was ranked in descending order. CpG sites ranked in top 10% in both discovery and validation sets were selected for further evaluation by Cox regression. Multiple testing correction by false discovery rate (FDR) method was used if necessary. (PDF 357 kb)

Additional file 5: **Figure S3.** DNA methylation probes identified by flexible criterion in squamous cell carcinoma and their correlation with corresponding gene expression. Three more sites were identified in SCC samples under flexible criterion: cg06646494 in *KDM2B*, cg00121158 in *KDM4C*, and cg06615743 in *KDM4B*. The cg00121158 (A) and cg06615743 (B) showed a statistical correlation with the corresponding gene expression. DNA methylation level was categorized to six quantiles and box plot for gene expression was drawn for each quantile. Pearson correlation was used to estimate the correlation coefficient (r) and the *P* value, Gene expression was log2 transformed before analysis. (PDF 571 kb)

## Abbreviations

95%CI: 95% confidence interval
AC: Lung adenocarcinomas
CART: Classification and regression tree
CV: Coefficient of variance
FDR: False discovery rate
*HR*: Hazard ratio
MGH: Massachusetts General Hospital
NSCLC: Non-small cell lung cancer
QC: Quality control
RF: Random forest
RSEM: RNA Sequencing by Expectation Maximization
SCC: Squamous cell carcinomas
SD: Standard deviation
TCGA: The Cancer Genome Atlas
TSS: Transcriptional start site
VIS: Variable importance scores

## References

[1] Siegel R, Ma J, Zou Z, Jemal A (2014). Cancer statistics, 2014. CA Cancer J Clin.

[2] Rami-Porta R, Asamura H, Travis WD, Rusch VW (2017). Lung cancer—major changes in the American Joint Committee on Cancer eighth edition cancer staging manual. CA Cancer J Clin.

[3] Tang S, Pan Y, Wang Y, Hu L, Cao S, Chu M, Dai J, Shu Y, Xu L, Chen J (2015). Genome-wide association study of survival in early-stage non-small cell lung cancer. Ann Surg Oncol.

[4] Heyn H, Esteller M (2012). DNA methylation profiling in the clinic: applications and challenges. Nat Rev Genet.

[5] Bannister AJ, Kouzarides T (2011). Regulation of chromatin by histone modifications. Cell Res.

[6] Thinnes CC, England KS, Kawamura A, Chowdhury R, Schofield CJ, Hopkinson RJ (1839). Targeting histone lysine demethylases—progress, challenges, and the future. Biochim Biophys Acta.

[7] Hojfeldt JW, Agger K, Helin K (2013). Histone lysine demethylases as targets for anticancer therapy. Nat Rev Drug Discov.

[8] Black JC, Whetstine JR (2013). Tipping the lysine methylation balance in disease. Biopolymers.

[9] Topper MJ, Vaz M, Chiappinelli KB, DeStefano Shields CE, Niknafs N, Yen RC, Wenzel A, Hicks J, Ballew M, Stone M (2017). Epigenetic therapy ties MYC depletion to reversing immune evasion and treating lung cancer. Cell.

[10] Kalin JH, Wu M, Gomez AV, Song Y, Das J, Hayward D, Adejola N, Wu M, Panova I, Chung HJ (2018). Targeting the CoREST complex with dual histone deacetylase and demethylase inhibitors. Nat Commun.

[11] Maes T, Mascaro C, Ortega A, Lunardi S, Ciceri F, Somervaille TC, Buesa C (2015). KDM1 histone lysine demethylases as targets for treatments of oncological and neurodegenerative disease. Epigenomics.

[12] Kong L, Zhang P, Li W, Yang Y, Tian Y, Wang X, Chen S, Yang Y, Huang T, Zhao T (2016). KDM1A promotes tumor cell invasion by silencing TIMP3 in non-small cell lung cancer cells. Oncotarget.

[13] Wagner KW, Alam H, Dhar SS, Giri U, Li N, Wei Y, Giri D, Cascone T, Kim JH, Ye Y (2013). KDM2A promotes lung tumorigenesis by epigenetically enhancing ERK1/2 signaling. J Clin Invest.

[14] Dhar SS, Alam H, Li N, Wagner KW, Chung J, Ahn YW, Lee MG (2014). Transcriptional repression of histone deacetylase 3 by the histone demethylase KDM2A is coupled to tumorigenicity of lung cancer cells. J Biol Chem.

[15] Black JC, Van Rechem C, Whetstine JR (2012). Histone lysine methylation dynamics: establishment, regulation, and biological impact. Mol Cell.

[16] Van Rechem C, Black JC, Greninger P, Zhao Y, Donado C, Burrowes PD, Ladd B, Christiani DC, Benes CH, Whetstine JR (2015). A coding single nucleotide polymorphism in lysine demethylase KDM4A associates with increased sensitivity to mTOR inhibitors. Cancer Discov.

[17] Van Rechem C, Black JC, Boukhali M, Aryee MJ, Graslund S, Haas W, Benes CH, Whetstine JR. Lysine demethylase KDM4A associates with translation machinery and regulates protein synthesis. Cancer Discov. 2015;5(3):255-63. 10.1158/2159-8290.CD-14-1326 PMC4355328 25564516

[18] Asomaning K, Miller DP, Liu G, Wain JC, Lynch TJ, Su L, Christiani DC (2008). Second hand smoke, age of exposure and lung cancer risk. Lung Cancer.

[19] Sandoval J, Mendez-Gonzalez J, Nadal E, Chen G, Carmona FJ, Sayols S, Moran S, Heyn H, Vizoso M, Gomez A (2013). A prognostic DNA methylation signature for stage I non-small-cell lung cancer. J Clin Oncol.

[20] Bjaanaes MM, Fleischer T, Halvorsen AR, Daunay A, Busato F, Solberg S, Jorgensen L, Kure E, Edvardsen H, Borresen-Dale AL (2016). Genome-wide DNA methylation analyses in lung adenocarcinomas: association with EGFR, KRAS and TP53 mutation status, gene expression and prognosis. Mol Oncol.

[21] Karlsson A, Jonsson M, Lauss M, Brunnstrom H, Jonsson P, Borg A, Jonsson G, Ringner M, Planck M, Staaf J (2014). Genome-wide DNA methylation analysis of lung carcinoma reveals one neuroendocrine and four adenocarcinoma epitypes associated with patient outcome. Clin Cancer Res.

[22] Chen YA, Lemire M, Choufani S, Butcher DT, Grafodatskaya D, Zanke BW, Gallinger S, Hudson TJ, Weksberg R (2013). Discovery of cross-reactive probes and polymorphic CpGs in the Illumina Infinium HumanMethylation450 microarray. Epigenetics.

[23] Marabita F, Almgren M, Lindholm ME, Ruhrmann S, Fagerstrom-Billai F, Jagodic M, Sundberg CJ, Ekstrom TJ, Teschendorff AE, Tegner J, Gomez-Cabrero D (2013). An evaluation of analysis pipelines for DNA methylation profiling using the Illumina HumanMethylation450 BeadChip platform. Epigenetics.

[24] Johnson WE, Li C, Rabinovic A (2007). Adjusting batch effects in microarray expression data using empirical Bayes methods. Biostatistics.

[25] Breiman L (2001). Random forests. Mach Learn.

[26] Wright MN, Ziegler A. Ranger: a fast implementation of random forests for high dimensional data in C++ and R. J Stat Softw. 2017;077:1–17.

[27] Baylin SB, Jones PA (2011). A decade of exploring the cancer epigenome—biological and translational implications. Nat Rev Cancer.

[28] Brenet F, Moh M, Funk P, Feierstein E, Viale AJ, Socci ND, Scandura JM (2011). DNA methylation of the first exon is tightly linked to transcriptional silencing. PLoS One.

[29] Lomelin D, Jorgenson E, Risch N (2010). Human genetic variation recognizes functional elements in noncoding sequence. Genome Res.

[30] Yang X, Han H, De Carvalho DD, Lay FD, Jones PA, Liang G (2014). Gene body methylation can alter gene expression and is a therapeutic target in cancer. Cancer Cell.

[31] Wagner JR, Busche S, Ge B, Kwan T, Pastinen T, Blanchette M (2014). The relationship between DNA methylation, genetic and expression inter-individual variation in untransformed human fibroblasts. Genome Biol.

[32] Maurano MT, Wang H, John S, Shafer A, Canfield T, Lee K, Stamatoyannopoulos JA (2015). Role of DNA methylation in modulating transcription factor occupancy. Cell Rep.

[33] Cancer Genome Atlas Research N (2012). Comprehensive genomic characterization of squamous cell lung cancers. Nature.

[34] Devarakonda S, Morgensztern D, Govindan R (2015). Genomic alterations in lung adenocarcinoma. Lancet Oncol.

[35] He J, Nguyen AT, Zhang Y (2011). KDM2b/JHDM1b, an H3K36me2-specific demethylase, is required for initiation and maintenance of acute myeloid leukemia. Blood.

[36] Tzatsos A, Paskaleva P, Ferrari F, Deshpande V, Stoykova S, Contino G, Wong KK, Lan F, Trojer P, Park PJ, Bardeesy N (2013). KDM2B promotes pancreatic cancer via Polycomb-dependent and -independent transcriptional programs. J Clin Invest.

[37] Kottakis F, Foltopoulou P, Sanidas I, Keller P, Wronski A, Dake BT, Ezell SA, Shen Z, Naber SP, Hinds PW (2014). NDY1/KDM2B functions as a master regulator of polycomb complexes and controls self-renewal of breast cancer stem cells. Cancer Res.

[38] Hong X, Xu Y, Qiu X, Zhu Y, Feng X, Ding Z, Zhang S, Zhong L, Zhuang Y, Su C (2016). MiR-448 promotes glycolytic metabolism of gastric cancer by downregulating KDM2B. Oncotarget.

[39] Berry WL, Janknecht R (2013). KDM4/JMJD2 histone demethylases: epigenetic regulators in cancer cells. Cancer Res.

[40] Black JC, Manning AL, Van Rechem C, Kim J, Ladd B, Cho J, Pineda CM, Murphy N, Daniels DL, Montagna C (2013). KDM4A lysine demethylase induces site-specific copy gain and rereplication of regions amplified in tumors. Cell.

[41] Young LC, McDonald DW, Hendzel MJ (2013). Kdm4b histone demethylase is a DNA damage response protein and confers a survival advantage following gamma-irradiation. J Biol Chem.

[42] Kim TD, Fuchs JR, Schwartz E, Abdelhamid D, Etter J, Berry WL, Li C, Ihnat MA, Li PK, Janknecht R (2014). Pro-growth role of the JMJD2C histone demethylase in HCT-116 colon cancer cells and identification of curcuminoids as JMJD2 inhibitors. Am J Transl Res.

[43] Zhao Y, Chen F, Zhai R, Lin X, Wang Z, Su L, Christiani DC (2012). Correction for population stratification in random forest analysis. Int J Epidemiol.

[44] Wu X, Gu J, Grossman HB, Amos CI, Etzel C, Huang M, Zhang Q, Millikan RE, Lerner S, Dinney CP (2006). Bladder cancer predisposition: a multigenic approach to DNA-repair and cell-cycle–control genes. Am J Hum Genet.

[45] Chen M, Kamat AM, Huang M, Grossman HB, Dinney CP, Lerner SP, Wu X, Gu J (2007). High-order interactions among genetic polymorphisms in nucleotide excision repair pathway genes and smoking in modulating bladder cancer risk. Carcinogenesis.

