# Supplementary figures and images for: Epigenetic modifications in KDM lysine demethylases associate with survival of early-stage NSCLC
Source: Clin Epigenetics. 2018 Apr 2;10:41. doi: 10.1186/s13148-018-0474-3 (PMC5879927; doi:10.1186/s13148-018-0474-3)

Figure S1

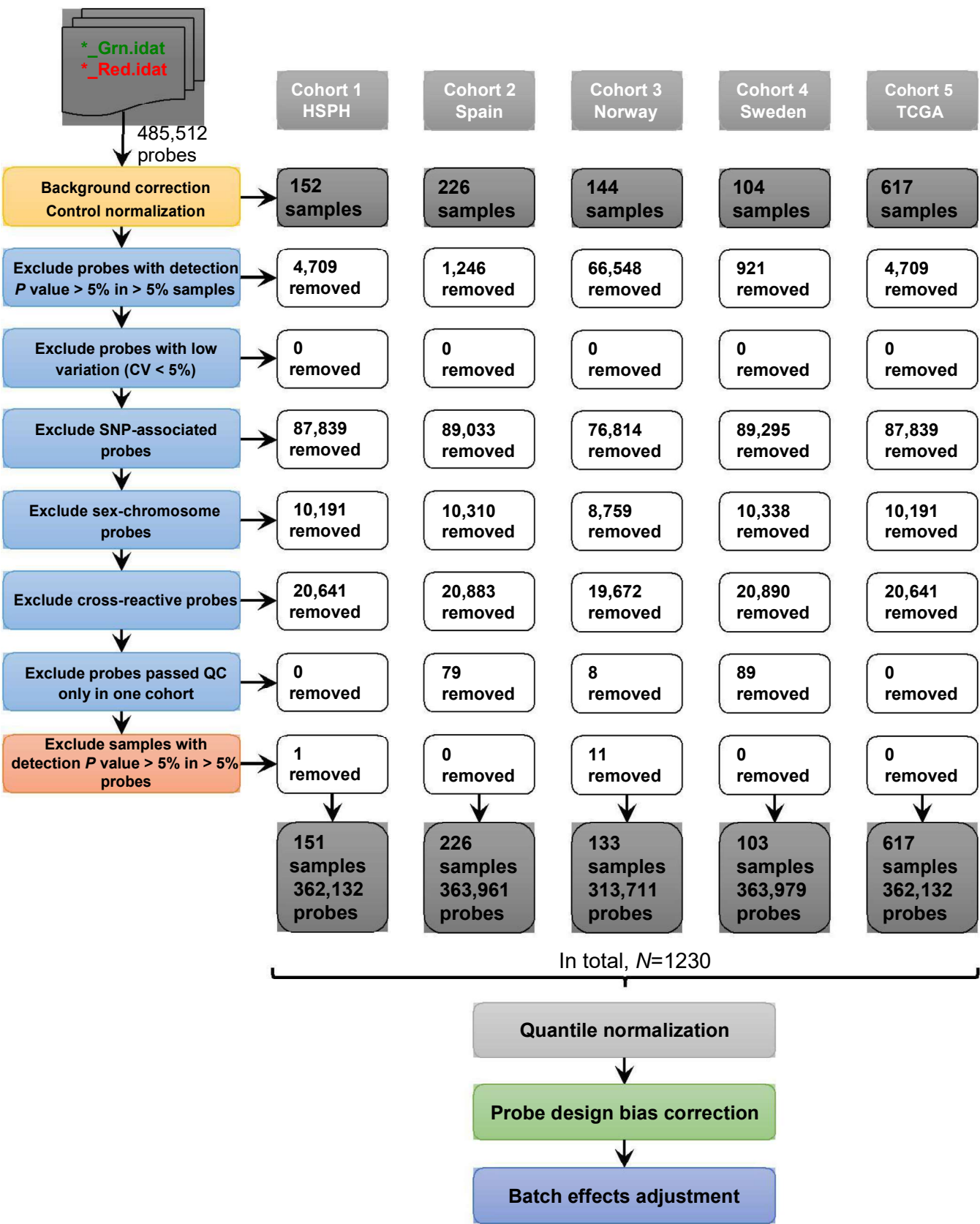

Supplement: Supplementary file 1 — Figure S1. Quality control processes for DNA methylation chip data. Quality control measures and exclusion criteria as applied to Harvard, Spain, Norway, Sweden, and The Cancer Genome Atlas (TCGA) sample cohorts. (PDF 642 kb) [file 13148_2018_474_MOESM1_ESM.pdf]

**Figure S2. Analysis work flow under flexible criteria**

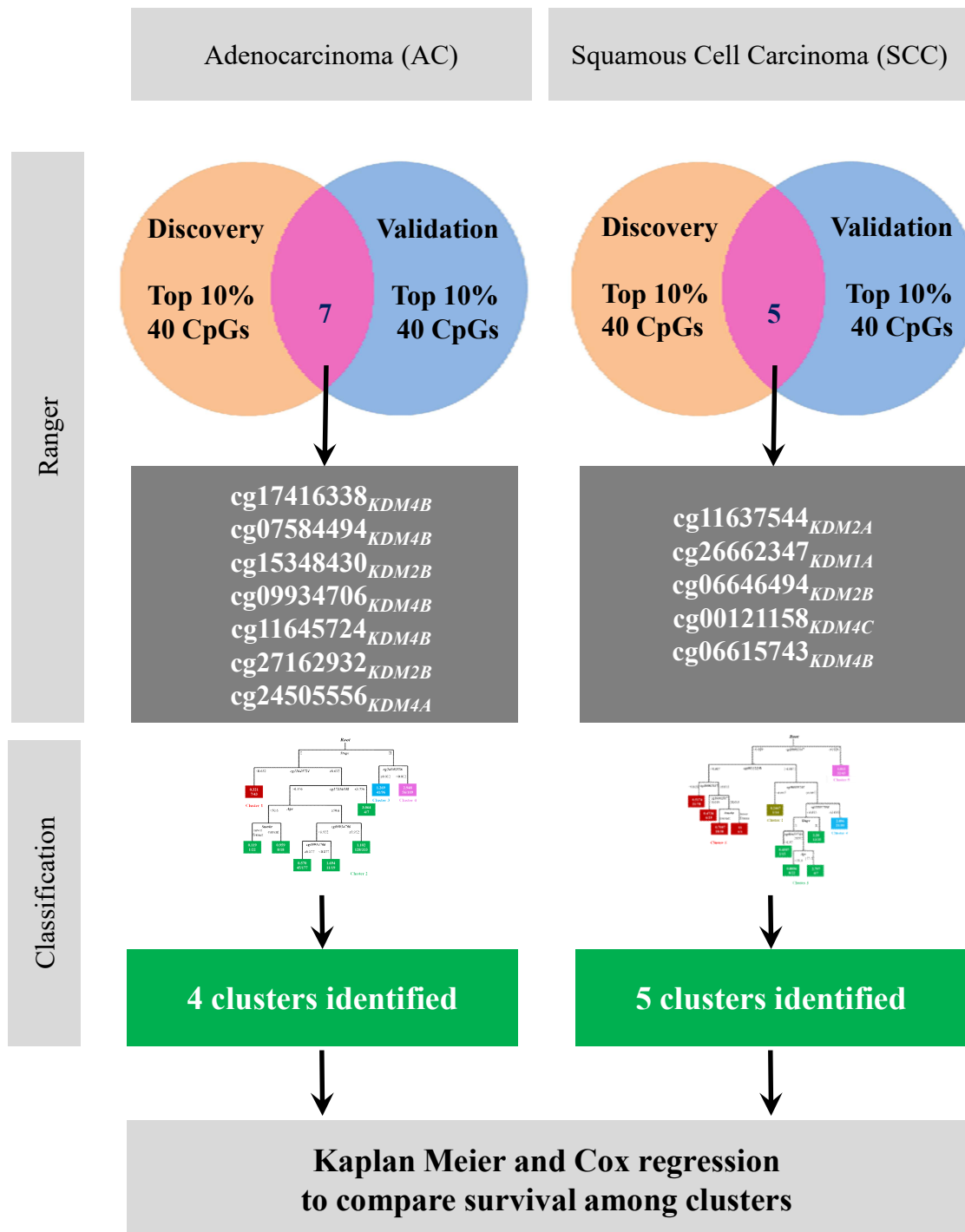

Supplement: Supplementary file 4 — Figure S2. Analysis work flow. Adenocarcinoma and squamous cell carcinoma samples from Harvard, Spain, Norway, and Sweden cohorts were used for the discovery phase of analysis. Data from The Cancer Genome Atlas (TCGA) were used for Validation. Ranger is a weighted version of random forest for controlling for the covariates including age, gender, smoking status, and histological stage. Variable importance score (VIS) was estimated for each CpG site and was ranked in descending order. CpG sites ranked in top 10% in both discovery and validation sets were selected for further evaluation by Cox regression. Multiple testing correction by false discovery rate (FDR) method was used if necessary. (PDF 357 kb) [file 13148_2018_474_MOESM4_ESM.pdf]
