# Supplementary material for: Epigenetic modifications in KDM lysine demethylases associate with survival of early-stage NSCLC
Source: Clin Epigenetics. 2018 Apr 2;10:41. doi: 10.1186/s13148-018-0474-3 (PMC5879927; doi:10.1186/s13148-018-0474-3)
Supplement: Supplementary file 2 — Table S1. Annotation of CpG sites in KDM gene family. (PDF 677 kb) [file 13148_2018_474_MOESM2_ESM.pdf]

Table S1: Annotation of CpG sites in KDM gene family

Seq	Probe	Infinium Design Type	CHR	Position	Gene Symbol	Domain	Relation_to CpG_Island	DMR	Enhancer	Feature_Group	DHS
1	cg00014203	I	17	7757969	KDM6B	3'UTR	N_Shore				
2	cg00031013	II	17	7757933	KDM6B	3'UTR	N_Shore				
3	cg00031896	I	12	122020205	KDM2B	TSS1500	S_Shore			Promoter_Associated	
4	cg00062312	II	1	23346344	KDM1A	1stExon	Island				
5	cg00102920	II	6	18156401	KDM1B	5'UTR	S_Shore			Promoter_Associated	
6	cg00121158	II	9	6757826	KDM4C	5'UTR	Island			Promoter_Associated	
7	cg00154982	I	1	44115597	KDM4A	TSS200	N_Shore			Promoter_Associated	
8	cg00265212	II	12	498607	KDM5A	5'UTR	Island			Promoter_Associated	
9	cg00435490	II	5	137687748	KDM3B	TSS1500	N_Shore			Promoter_Associated	
10	cg00513611	II	19	5061205	KDM4B	Body	Island				
11	cg00528052	II	1	202777295	KDM5B	1stExon	Island				TRUE
12	cg00529909	II	19	5108734	KDM4B	Body	N_Shore			Unclassified_Cell_type_specific	
13	cg00541565	I	1	23346403	KDM1A	1stExon	Island				
14	cg00571448	II	1	202776680	KDM5B	Body	Island				TRUE
15	cg00636124	I	16	27215281	KDM8	5'UTR	Island			Promoter_Associated	
16	cg00667760	II	9	6757146	KDM4C	TSS1500	N_Shore			Promoter_Associated	
17	cg00707814	II	12	121893668	KDM2B	Body	S_Shelf				
18	cg00867626	I	12	121905749	KDM2B	Body	Island				
19	cg00946598	II	1	44117093	KDM4A	5'UTR	S_Shore			Promoter_Associated_ Cell_type_specific	
20	cg00979347	II	1	44115798	KDM4A	1stExon	Island			Promoter_Associated	
21	cg01029623	II	12	122016779	KDM2B	Body	Island	DMR			TRUE
22	cg01046511	II	17	7742971	KDM6B	TSS1500	N_Shelf		TRUE		
23	cg01047218	II	11	67007568	KDM2A	Body				Promoter_Associated	
24	cg01068259	II	12	121975344	KDM2B	Body	Island			Promoter_Associated	
25	cg01098771	I	19	4969087	KDM4B	TSS200	Island			Promoter_Associated	
26	cg01154721	I	12	121881891	KDM2B	Body	N_Shore			Gene_Associated	
27	cg01214587	II	12	121891131	KDM2B	Body	Island				
28	cg01504836	I	11	94706833	KDM4D	TSS200	Island			Promoter_Associated	
29	cg01530797	II	7	139875863	KDM7A	Body	Island				
30	cg01543795	I	16	27215147	KDM8	5'UTR	Island			Promoter_Associated	
31	cg01547069	I	7	139877097	KDM7A	Body	Island			Promoter_Associated	

Table S1: Annotation of CpG sites in KDM gene family

Seq	Probe	Infinium Design Type	CHR	Position	Gene Symbol	Domain	Relation_to CpG_Island	DMR	Enhancer	Feature_Group	DHS
32	cg01640991	I	12	121947315	KDM2B	Body					
33	cg01663745	I	11	94706857	KDM4D	1stExon	Island			Promoter_Associated	
34	cg01744130	I	2	86667850	KDM3A	TSS1500	Island			Promoter_Associated	TRUE
35	cg01755539	I	16	27215246	KDM8	5'UTR	Island			Promoter_Associated	
36	cg01975510	II	11	67017821	KDM2A	Body	Island				
37	cg02152982	II	2	86667766	KDM3A	TSS1500	N_Shore			Promoter_Associated	TRUE
38	cg02182114	II	7	139876578	KDM7A	TSS1500	Island				
39	cg02302043	I	19	4988178	KDM4B	5'UTR	S_Shore		TRUE		
40	cg02308232	II	17	7742762	KDM6B	TSS1500	N_Shelf		TRUE	Unclassified_Cell_type_ specific	
41	cg02331272	II	11	66964208	KDM2A	Body			TRUE		
42	cg02340915	I	12	121904480	KDM2B	Body	N_Shore				
43	cg02386983	II	2	86669112	KDM3A	5'UTR	Island				
44	cg02411582	II	16	27232615	KDM8	3'UTR					
45	cg02491696	II	12	498417	KDM5A	5'UTR	Island			Promoter_Associated	
46	cg02577963	I	19	5041570	KDM4B	Body	N_Shore			Unclassified_Cell_type_specific	
47	cg02590486	II	12	499376	KDM5A	TSS1500	S_Shore			Promoter_Associated	
48	cg02606627	I	19	5047711	KDM4B	Body	Island				
49	cg02633409	II	19	5051110	KDM4B	Body	S_Shelf			Unclassified_Cell_type_specific	
50	cg02659867	II	19	5033098	KDM4B	Body	Island			Unclassified	
51	cg02712585	II	19	5142260	KDM4B	Body	Island			Unclassified_Cell_type_specific	
52	cg02801993	II	19	5062235	KDM4B	Body	S_Shore				
53	cg02827029	I	12	121890907	KDM2B	Body	Island				
54	cg02842850	I	19	5148130	KDM4B	Body	Island				TRUE
55	cg02871891	II	16	27228679	KDM8	Body					TRUE
56	cg02903589	II	6	18215340	KDM1B	Body					
57	cg02911492	II	11	67017791	KDM2A	Body	Island				
58	cg02950740	II	12	498876	KDM5A	TSS1500	Island			Promoter_Associated	
59	cg02972551	II	2	86668068	KDM3A	TSS1500	Island			Promoter_Associated	TRUE
60	cg03037033	I	2	86668736	KDM3A	5'UTR	Island				TRUE
61	cg03101936	II	16	27227277	KDM8	Body					
62	cg03241244	I	12	122017052	KDM2B	Body	Island	DMR		Promoter_Associated_Cell_type_specific	TRUE

Table S1: Annotation of CpG sites in KDM gene family

Seq	Probe	Infinium Design Type	CHR	Position	Gene Symbol	Domain	Relation_to CpG_Island	DMR	Enhancer	Feature_Group	DHS
63	cg03310779	II	19	5117559	KDM4B	Body	N_Shore				
64	cg03315484	I	5	137688433	KDM3B	1stExon	Island			Promoter_Associated	
65	cg03315649	II	19	5093541	KDM4B	Body	Island				
66	cg03358468	II	19	5060985	KDM4B	Body	Island				
67	cg03389662	I	11	67007586	KDM2A	Body				Promoter_Associated	
68	cg03486832	II	12	122019525	KDM2B	TSS1500	S_Shore	RDMR		Promoter_Associated	
69	cg03538808	II	17	7746830	KDM6B	5'UTR	Island			Promoter_Associated	
70	cg03556944	II	19	5142391	KDM4B	Body	Island			Unclassified_Cell_type_specific	
71	cg03587511	II	1	44115742	KDM4A	TSS200	Island			Promoter_Associated	
72	cg03592906	II	19	5146141	KDM4B	Body	Island				
73	cg03607513	II	11	94707155	KDM4D	1stExon	S_Shore			Promoter_Associated	
74	cg03688668	I	19	5065905	KDM4B	Body	Island				
75	cg03719978	I	17	7758102	KDM6B	3'UTR	N_Shore				
76	cg03727342	II	9	6758683	KDM4C	5'UTR	Island				
77	cg03854722	II	17	7757958	KDM6B	3'UTR	N_Shore				
78	cg04085336	II	11	67007785	KDM2A	Body				Promoter_Associated	
79	cg04231636	II	6	18186411	KDM1B	Body			TRUE		
80	cg04241305	II	5	137689053	KDM3B	Body	Island				
81	cg04384209	II	19	5029938	KDM4B	5'UTR	N_Shelf			Unclassified	TRUE
82	cg04388472	II	11	94706739	KDM4D	TSS200	Island			Promoter_Associated	
83	cg04406115	II	19	5065640	KDM4B	Body	Island				
84	cg04504182	I	1	202777459	KDM5B	1stExon	Island				TRUE
85	cg04585679	II	19	5044863	KDM4B	Body	S_Shore			Unclassified_Cell_type_specific	
86	cg04586041	I	12	121878382	KDM2B	Body	N_Shore				
87	cg04592958	I	19	5138093	KDM4B	Body	N_Shelf			Unclassified_Cell_type_specific	TRUE
88	cg04615850	II	19	5048814	KDM4B	Body	Island			Unclassified_Cell_type_specific	
89	cg04682193	II	12	121867725	KDM2B	3'UTR					
90	cg04793821	I	2	86668538	KDM3A	5'UTR	Island			Promoter_Associated	TRUE
91	cg05038268	I	11	66885281	KDM2A	TSS1500	Island				
92	cg05067991	I	12	122018586	KDM2B	Body	S_Shore				TRUE
93	cg05191700	I	5	137688423	KDM3B	1stExon	Island			Promoter_Associated	
94	cg05295919	II	11	66886253	KDM2A	TSS1500	Island			Promoter_Associated	

Table S1: Annotation of CpG sites in KDM gene family

Seq	Probe	Infinium Design Type	CHR	Position	Gene Symbol	Domain	Relation_to CpG_Island	DMR	Enhancer	Feature_Group	DHS
95	cg05545111	II	9	6758140	KDM4C	5'UTR	Island			Promoter_Associated	
96	cg05557291	I	12	121958763	KDM2B	Body					
97	cg05708550	II	5	137688227	KDM3B	TSS200	Island			Promoter_Associated	
98	cg05713782	I	11	94706830	KDM4D	TSS200	Island			Promoter_Associated	
99	cg05722427	II	5	137688090	KDM3B	TSS200	N_Shore			Promoter_Associated	
100	cg05743713	I	11	66885240	KDM2A	TSS1500	N_Shore				
101	cg05877783	I	19	5096685	KDM4B	Body	Island				
102	cg05886813	II	1	44115187	KDM4A	TSS1500	N_Shore			Promoter_Associated	
103	cg05913962	II	16	27214514	KDM8	TSS1500	N_Shore			Promoter_Associated	
104	cg06024930	I	12	121890328	KDM2B	Body	Island	DMR			TRUE
105	cg06267927	I	12	498162	KDM5A	1stExon	Island			Promoter_Associated	
106	cg06320126	I	11	66886141	KDM2A	TSS1500	Island				
107	cg06329197	II	16	27214832	KDM8	1stExon	Island			Promoter_Associated	
108	cg06372176	II	17	7748321	KDM6B	5'UTR	Island		TRUE	Promoter_Associated	
109	cg06596689	I	7	139875956	KDM7A	TSS1500	Island				
110	cg06615743	II	19	5119574	KDM4B	Body	S_Shore				
111	cg06625677	I	17	7745595	KDM6B	5'UTR	Island			Promoter_Associated	
112	cg06627361	II	12	498468	KDM5A	5'UTR	Island			Promoter_Associated	
113	cg06646494	I	12	121882415	KDM2B	Body	Island				
114	cg06884029	II	19	4986305	KDM4B	5'UTR	Island				
115	cg06890028	II	1	44137602	KDM4A	Body			TRUE		TRUE
116	cg06941557	I	17	7757543	KDM6B	3'UTR	Island				
117	cg06958034	II	1	23346203	KDM1A	1stExon	Island				
118	cg06996029	I	11	67017617	KDM2A	Body	Island				
119	cg07020001	II	12	122019031	KDM2B	TSS200	S_Shore	RDMR		Promoter_Associated	
120	cg07052513	I	2	86668451	KDM3A	5'UTR	Island			Promoter_Associated	TRUE
121	cg07118078	II	1	23345725	KDM1A	TSS1500	N_Shore			Promoter_Associated	
122	cg07156959	I	12	121975669	KDM2B	Body	Island				
123	cg07165616	II	12	498868	KDM5A	TSS1500	Island			Promoter_Associated	
124	cg07211384	I	2	86668246	KDM3A	TSS1500	Island			Promoter_Associated	TRUE
125	cg07271268	II	12	498129	KDM5A	1stExon	Island			Promoter_Associated	
126	cg07283932	I	11	66887736	KDM2A	5'UTR	S_Shore				

Table S1: Annotation of CpG sites in KDM gene family

Seq	Probe	Infinium Design Type	CHR	Position	Gene Symbol	Domain	Relation_to CpG_Island	DMR	Enhancer	Feature_Group	DHS
127	cg07361448	II	16	27230286	KDM8	Body					
128	cg07474126	I	12	121975243	KDM2B	Body	Island			Promoter_Associated	
129	cg07482916	II	17	7757646	KDM6B	3'UTR	Island				
130	cg07529210	I	12	122019110	KDM2B	TSS200	S_Shore	RDMR		Promoter_Associated	
131	cg07584494	I	19	5074758	KDM4B	Body	Island				
132	cg07741840	II	12	121893769	KDM2B	Body	S_Shelf				
133	cg07785945	I	1	23346356	KDM1A	1stExon	Island				
134	cg07793808	II	12	122019006	KDM2B	TSS200	S_Shore	RDMR			
135	cg07801662	II	17	7745785	KDM6B	5'UTR	Island			Promoter_Associated	
136	cg07846737	I	19	5068701	KDM4B	Body	S_Shelf				
137	cg08036796	II	19	5034282	KDM4B	Body	S_Shore				
138	cg08043998	II	12	121890503	KDM2B	Body	Island				TRUE
139	cg08180261	I	2	86668980	KDM3A	5'UTR	Island				TRUE
140	cg08355583	II	7	139875917	KDM7A	TSS1500	Island				
141	cg08461425	II	12	121971790	KDM2B	Body	N_Shelf		TRUE		
142	cg08462360	II	1	44119109	KDM4A	Body	S_Shelf				
143	cg08520742	II	12	121882033	KDM2B	Body	N_Shore			Gene_Associated	
144	cg08572679	I	16	27214781	KDM8	TSS1500	Island			Promoter_Associated	
145	cg08632473	II	6	18221097	KDM1B	Body			TRUE		
146	cg08701479	I	19	5135291	KDM4B	Body	Island				
147	cg08836144	II	11	66886918	KDM2A	1stExon	Island			Promoter_Associated	
148	cg08840665	I	17	7756882	KDM6B	3'UTR	Island				
149	cg08915090	I	1	202777584	KDM5B	TSS200	Island				TRUE
150	cg09074260	II	11	94707049	KDM4D	1stExon	Island			Promoter_Associated	
151	cg09140090	I	16	27215225	KDM8	5'UTR	Island			Promoter_Associated	
152	cg09151742	II	12	122018897	KDM2B	5'UTR	S_Shore				
153	cg09175485	II	11	67020555	KDM2A	Body	S_Shelf			Gene_Associated	
154	cg09310112	II	19	4969989	KDM4B	5'UTR	S_Shore				
155	cg09411874	II	12	121905955	KDM2B	Body	Island				
156	cg09482780	I	17	7756609	KDM6B	Body	Island				
157	cg09539800	I	7	139877104	KDM7A	Body	Island			Promoter_Associated	
158	cg09544705	II	11	67007628	KDM2A	Body				Promoter_Associated	

Table S1: Annotation of CpG sites in KDM gene family

Seq	Probe	Infinium Design Type	CHR	Position	Gene Symbol	Domain	Relation_to CpG_Island	DMR	Enhancer	Feature_Group	DHS
159	cg09632858	II	16	27219548	KDM8	Body	S_Shelf				
160	cg09853387	II	7	139877374	KDM7A	Body	Island				
161	cg09934706	II	19	5147855	KDM4B	Body	Island			Unclassified_Cell_type_specific	
162	cg10124355	II	19	5037004	KDM4B	Body	Island		TRUE		TRUE
163	cg10208587	II	17	7756573	KDM6B	Body	Island				
164	cg10214171	II	16	27215034	KDM8	TSS1500	Island			Promoter_Associated	
165	cg10214350	II	17	7747091	KDM6B	5'UTR	Island			Promoter_Associated	
166	cg10221365	II	16	27214422	KDM8	TSS1500	N_Shore			Promoter_Associated	
167	cg10499166	II	1	23345815	KDM1A	TSS200	Island			Promoter_Associated	
168	cg10612065	II	19	5135544	KDM4B	Body	Island				
169	cg10704534	II	12	121882208	KDM2B	Body	Island			Gene_Associated	
170	cg10717766	I	19	5134118	KDM4B	Body	Island				
171	cg10765877	II	12	121973425	KDM2B	Body	N_Shore	RDMR		Promoter_Associated_Cell_type_specific	
172	cg10821320	II	19	5151204	KDM4B	Body	N_Shore		TRUE	Unclassified_Cell_type_specific	
173	cg10968649	II	1	44115804	KDM4A	1stExon	Island			Promoter_Associated	
174	cg11007366	I	6	18155579	KDM1B	TSS200	Island			Promoter_Associated	
175	cg11214164	II	12	498496	KDM5A	5'UTR	Island			Promoter_Associated	
176	cg11221497	I	1	202776298	KDM5B	Body	Island				TRUE
177	cg11225935	II	12	498478	KDM5A	5'UTR	Island			Promoter_Associated	
178	cg11378778	I	19	5144334	KDM4B	Body	Island				
179	cg11637544	II	11	66886765	KDM2A	1stExon	Island			Promoter_Associated	
180	cg11645724	II	19	5072741	KDM4B	Body	N_Shore				
181	cg11724984	I	12	121890864	KDM2B	Body	Island				
182	cg11782515	II	19	4986183	KDM4B	5'UTR	Island				
183	cg11837965	II	19	5096856	KDM4B	Body	Island				
184	cg12069073	II	11	67007553	KDM2A	Body				Promoter_Associated	
185	cg12072803	II	19	4968558	KDM4B	TSS1500	Island			Promoter_Associated	
186	cg12077818	II	1	44116508	KDM4A	5'UTR	S_Shore			Promoter_Associated	
187	cg12099191	II	19	5100020	KDM4B	Body	S_Shelf			Unclassified_Cell_type_specific	
188	cg12251659	I	12	122018385	KDM2B	TSS200	Island				TRUE
189	cg12293275	II	1	44115487	KDM4A	TSS1500	N_Shore			Promoter_Associated	

Table S1: Annotation of CpG sites in KDM gene family

Seq	Probe	Infinium Design Type	CHR	Position	Gene Symbol	Domain	Relation_to CpG_Island	DMR	Enhancer	Feature_Group	DHS
190	cg12352601	II	12	121947522	KDM2B	Body					
191	cg12578844	I	1	23346212	KDM1A	1stExon	Island				
192	cg12639558	II	12	121958832	KDM2B	Body					
193	cg12800105	II	12	121972412	KDM2B	Body	N_Shelf	CDMR		Unclassified	
194	cg12869958	I	11	67017687	KDM2A	Body	Island				
195	cg12872398	II	19	5110601	KDM4B	Body	Island				
196	cg12888358	II	2	86668281	KDM3A	5'UTR	Island			Promoter_Associated	TRUE
197	cg12888660	II	12	122018449	KDM2B	TSS200	Island				TRUE
198	cg13149662	I	12	122017211	KDM2B	Body	Island				TRUE
199	cg13253785	II	1	202776269	KDM5B	Body	Island				
200	cg13278445	I	17	7745945	KDM6B	5'UTR	Island			Promoter_Associated	
201	cg13357776	II	19	5110616	KDM4B	Body	Island				
202	cg13456960	II	19	5139479	KDM4B	Body	N_Shore			Unclassified_Cell_type_specific	TRUE
203	cg13517826	II	19	5039857	KDM4B	Body	Island				
204	cg13559217	I	19	5119331	KDM4B	Body	Island				
205	cg13656025	I	17	7744978	KDM6B	5'UTR	N_Shore			Promoter_Associated	
206	cg13708645	II	12	121974305	KDM2B	Body	N_Shore		TRUE	Promoter_Associated	
207	cg13743500	I	9	6761072	KDM4C	5'UTR	S_Shelf				
208	cg13842202	II	9	7172362	KDM4C	Body			TRUE		
209	cg13846232	II	17	7752867	KDM6B	Body	N_Shelf				
210	cg13859589	II	9	7148129	KDM4C	Body					
211	cg13875807	II	9	6758935	KDM4C	5'UTR	S_Shore				
212	cg14010696	II	19	5119250	KDM4B	Body	Island				
213	cg14015920	II	1	23346770	KDM1A	Body	S_Shore				
214	cg14041232	II	9	6921930	KDM4C	Body					
215	cg14090211	II	19	5077526	KDM4B	Body	S_Shore				
216	cg14138312	II	9	6869699	KDM4C	Body			TRUE		
217	cg14180502	I	9	6758546	KDM4C	5'UTR	Island				
218	cg14196303	II	9	6757347	KDM4C	TSS1500	Island			Promoter_Associated	
219	cg14381677	II	1	202778618	KDM5B	TSS1500	N_Shore				
220	cg14473811	II	9	6757912	KDM4C	5'UTR	Island			Promoter_Associated	

Table S1: Annotation of CpG sites in KDM gene family

Seq	Probe	Infinium Design Type	CHR	Position	Gene Symbol	Domain	Relation_to CpG_Island	DMR	Enhancer	Feature_Group	DHS
221	cg14489470	II	12	121880054	KDM2B	Body	Island				
222	cg14497348	I	9	6757781	KDM4C	5'UTR	Island			Promoter_Associated	
223	cg14580919	II	19	5141553	KDM4B	Body	Island			Unclassified_Cell_type_specific	TRUE
224	cg14674091	II	11	94756993	KDM4E	TSS1500					
225	cg14694038	II	19	5037103	KDM4B	Body	Island		TRUE		TRUE
226	cg14791922	II	19	5144160	KDM4B	Body	Island			Unclassified	
227	cg14804129	I	19	5110798	KDM4B	Body	Island				
228	cg14963860	II	11	94706164	KDM4D	5'UTR	N_Shore			Promoter_Associated	
229	cg15001364	I	5	137688694	KDM3B	Body	Island			Promoter_Associated	
230	cg15120237	II	17	7747620	KDM6B	5'UTR	Island		TRUE		
231	cg15121288	II	11	67007855	KDM2A	Body				Promoter_Associated	
232	cg15124342	I	6	18155283	KDM1B	TSS1500	N_Shore			Promoter_Associated	
233	cg15174682	I	19	5132063	KDM4B	Body	N_Shore		TRUE	Unclassified_Cell_type_specific	TRUE
234	cg15213650	II	19	4984614	KDM4B	5'UTR	N_Shore				
235	cg15234492	II	12	122019076	KDM2B	TSS200	S_Shore	RDMR		Promoter_Associated	
236	cg15237644	II	12	121878287	KDM2B	Body	N_Shore				
237	cg15316703	I	12	122018177	KDM2B	Body	Island			Promoter_Associated	TRUE
238	cg15340887	I	12	121904973	KDM2B	Body	N_Shore				
239	cg15348430	I	12	121905538	KDM2B	Body	Island				
240	cg15403654	I	17	7747012	KDM6B	5'UTR	Island			Promoter_Associated	
241	cg15555590	II	11	94758366	KDM4E	TSS200					
242	cg15589689	II	1	44170389	KDM4A	3'UTR	N_Shelf				
243	cg15695155	II	12	121973871	KDM2B	Body	N_Shore	RDMR	TRUE		
244	cg15695435	II	12	121909465	KDM2B	Body	S_Shelf		TRUE		
245	cg15789385	II	12	121907156	KDM2B	Body	S_Shore				
246	cg15842967	II	11	67007584	KDM2A	Body				Promoter_Associated	
247	cg16004548	II	11	66887657	KDM2A	5'UTR	S_Shore				
248	cg16049335	II	17	7748444	KDM6B	5'UTR	S_Shore			Promoter_Associated	
249	cg16320626	I	12	121877851	KDM2B	Body	N_Shelf				
250	cg16617758	I	19	5114202	KDM4B	Body	S_Shelf		TRUE		
251	cg16636182	I	19	5112710	KDM4B	Body	S_Shore		TRUE		

Table S1: Annotation of CpG sites in KDM gene family

Seq	Probe	Infinium Design Type	CHR	Position	Gene Symbol	Domain	Relation_to CpG_Island	DMR	Enhancer	Feature_Group	DHS
252	cg16673806	I	5	137771872	KDM3B	3'UTR					
253	cg16752029	II	16	27214750	KDM8	TSS1500	N_Shore			Promoter_Associated	
254	cg16837338	II	12	122018770	KDM2B	1stExon	S_Shore				
255	cg16886828	I	19	5032663	KDM4B	5'UTR	Island			Unclassified	
256	cg16957323	II	17	7750695	KDM6B	Body	S_Shelf				
257	cg16993220	I	11	94706705	KDM4D	TSS200	Island			Promoter_Associated	
258	cg17146617	II	12	121883333	KDM2B	Body	S_Shore				
259	cg17169037	II	12	122015596	KDM2B	Body	N_Shore	RDMR	TRUE		TRUE
260	cg17339794	II	19	5074224	KDM4B	Body	N_Shore			Unclassified_Cell_type_specific	
261	cg17416338	II	19	5074717	KDM4B	Body	Island				
262	cg17452615	II	12	122019080	KDM2B	TSS200	S_Shore	RDMR		Promoter_Associated	
263	cg17711535	I	7	139877050	KDM7A	TSS1500	Island			Promoter_Associated	
264	cg17767427	II	19	5036971	KDM4B	Body	Island		TRUE		TRUE
265	cg17773654	II	19	5136588	KDM4B	Body	S_Shore				
266	cg17823829	II	1	202765754	KDM5B	Body			TRUE		
267	cg17933295	II	19	5076032	KDM4B	Body	Island				
268	cg18115693	II	19	4968519	KDM4B	TSS1500	N_Shore			Promoter_Associated	
269	cg18132081	II	11	66887329	KDM2A	1stExon	Island			Promoter_Associated	
270	cg18247177	II	17	7745472	KDM6B	5'UTR	Island			Promoter_Associated	
271	cg18362538	II	19	5032862	KDM4B	5'UTR	Island				
272	cg18473234	II	19	5097819	KDM4B	Body	S_Shore			Unclassified_Cell_type_specific	
273	cg18611949	II	2	86668510	KDM3A	5'UTR	Island			Promoter_Associated	TRUE
274	cg18682923	II	5	137687713	KDM3B	TSS1500	N_Shore			Promoter_Associated	
275	cg18795232	I	12	121890163	KDM2B	Body	N_Shore				TRUE
276	cg18836486	II	19	5070723	KDM4B	Body	N_Shelf				
277	cg18864768	II	1	44170024	KDM4A	Body	N_Shelf				
278	cg18865419	II	19	5064019	KDM4B	Body	N_Shore				
279	cg19076659	II	5	137688057	KDM3B	TSS1500	N_Shore			Promoter_Associated	
280	cg19139691	I	2	86668469	KDM3A	5'UTR	Island			Promoter_Associated	TRUE
281	cg19239104	I	19	5036609	KDM4B	Body	N_Shore				TRUE
282	cg19313221	II	19	5046567	KDM4B	Body	N_Shore				TRUE
283	cg19449286	I	17	7756708	KDM6B	Body	Island				

Table S1: Annotation of CpG sites in KDM gene family

Seq	Probe	Infinium Design Type	CHR	Position	Gene Symbol	Domain	Relation_to CpG_Island	DMR	Enhancer	Feature_Group	DHS
284	cg19499224	II	19	5146074	KDM4B	Body	Island				
285	cg19608357	I	19	5031251	KDM4B	5'UTR	N_Shore		TRUE	Unclassified	
286	cg19725989	I	11	67007604	KDM2A	Body				Promoter_Associated	
287	cg19743044	II	1	44115203	KDM4A	TSS1500	N_Shore			Promoter_Associated	
288	cg19764940	II	19	5141399	KDM4B	Body	Island			Unclassified_Cell_type_specific TRUE	
289	cg19839382	II	19	5036857	KDM4B	Body	N_Shore		TRUE		TRUE
290	cg20055546	I	12	122018463	KDM2B	TSS200	S_Shore				TRUE
291	cg20096208	II	11	94706512	KDM4D	5'UTR	Island			Promoter_Associated	
292	cg20098875	II	19	5093370	KDM4B	Body	N_Shore			Unclassified_Cell_type_specific	
293	cg20191795	II	2	86668233	KDM3A	TSS1500	Island			Promoter_Associated	TRUE
294	cg20320472	II	11	67025026	KDM2A	3'UTR					
295	cg20347343	II	19	5119355	KDM4B	Body	Island				
296	cg20395646	I	12	121947433	KDM2B	Body					
297	cg20677365	II	6	18155916	KDM1B	TSS1500	Island			Promoter_Associated	
298	cg20755408	II	12	498145	KDM5A	1stExon	Island			Promoter_Associated	
299	cg20873046	I	1	44115663	KDM4A	TSS200	Island			Promoter_Associated	
300	cg20878780	I	12	121880280	KDM2B	Body	Island				
301	cg20918903	II	2	86668501	KDM3A	5'UTR	Island			Promoter_Associated	TRUE
302	cg20942219	II	1	44141161	KDM4A	Body				Gene_Associated_Cell_type_specific	
303	cg20951334	II	2	86669446	KDM3A	Body	Island				
304	cg21126573	II	17	7755257	KDM6B	Body	Island		TRUE	Promoter_Associated	
305	cg21249371	I	12	122019117	KDM2B	TSS200	S_Shore	RDMR		Promoter_Associated	
306	cg21293464	II	11	66885276	KDM2A	TSS1500	Island				
307	cg21451504	I	1	44116235	KDM4A	5'UTR	Island			Promoter_Associated	
308	cg21526150	II	7	139875415	KDM7A	Body	Island			Promoter_Associated	
309	cg21580016	II	12	121887392	KDM2B	Body	N_Shelf				
310	cg21711868	I	11	66886748	KDM2A	1stExon	Island			Promoter_Associated	
311	cg21781916	I	12	122018375	KDM2B	TSS200	Island				TRUE
312	cg21973527	II	19	5090962	KDM4B	Body	N_Shelf		TRUE		
313	cg22079102	I	19	5061085	KDM4B	Body	Island				
314	cg22094309	II	1	44116120	KDM4A	5'UTR	Island			Promoter_Associated	
315	cg22127773	I	17	7754785	KDM6B	Body	N_Shore			Promoter_Associated	

Table S1: Annotation of CpG sites in KDM gene family

Seq	Probe	Infinium Design Type	CHR	Position	Gene Symbol	Domain	Relation_to CpG_Island	DMR	Enhancer	Feature_Group	DHS
316	cg22241443	II	11	67018220	KDM2A	Body	S_Shore				
317	cg22404242	II	2	86668131	KDM3A	TSS1500	Island			Promoter_Associated	TRUE
318	cg22406543	II	6	18155584	KDM1B	TSS200	Island			Promoter_Associated	
319	cg22646782	II	19	5152272	KDM4B	3'UTR	S_Shore			Unclassified_Cell_type_specific	
320	cg22997601	II	19	5115624	KDM4B	Body	N_Shelf				
321	cg23023599	II	11	94732500	KDM4D	3'UTR					
322	cg23073439	I	6	18155296	KDM1B	TSS1500	N_Shore			Promoter_Associated	
323	cg23485436	I	19	5048645	KDM4B	Body	Island			Unclassified_Cell_type_specific	
324	cg23771088	II	12	121975533	KDM2B	Body	Island			Promoter_Associated	
325	cg23838943	II	19	5049135	KDM4B	Body	S_Shore			Unclassified_Cell_type_specific	
326	cg23839891	I	19	5094994	KDM4B	Body	N_Shore				
327	cg23860886	I	6	18155593	KDM1B	TSS200	Island			Promoter_Associated	
328	cg23877401	I	12	122017247	KDM2B	Body	Island				TRUE
329	cg23879263	II	12	121978205	KDM2B	Body	S_Shelf				
330	cg23972735	I	12	121890311	KDM2B	Body	Island	DMR			TRUE
331	cg24035682	I	12	121958802	KDM2B	Body					
332	cg24109744	II	19	5107661	KDM4B	Body	N_Shelf				
333	cg24128630	II	17	7757287	KDM6B	3'UTR	Island			Unclassified_Cell_type_specific	
334	cg24137987	I	5	137688255	KDM3B	TSS200	Island			Promoter_Associated	
335	cg24206694	II	16	27214687	KDM8	TSS1500	N_Shore			Promoter_Associated	
336	cg24214903	II	19	5093659	KDM4B	Body	Island				
337	cg24230102	II	11	94760534	KDM4E	3'UTR				Unclassified_Cell_type_specific	
338	cg24236839	II	19	5038822	KDM4B	Body	N_Shore			TRUE	
339	cg24324755	I	1	44115813	KDM4A	1stExon	Island			Promoter_Associated	
340	cg24342628	II	6	18155101	KDM1B	TSS1500	N_Shore			Promoter_Associated	
341	cg24354901	I	1	202777590	KDM5B	TSS200	Island				TRUE
342	cg24400517	II	7	139876520	KDM7A	TSS1500	Island			Unclassified_Cell_type_specific	
343	cg24450508	I	19	5141352	KDM4B	Body	Island			TRUE	
344	cg24462596	I	11	94706862	KDM4D	1stExon	Island			Promoter_Associated	
345	cg24505556	I	1	44115737	KDM4A	TSS200	Island			Promoter_Associated	
346	cg24616466	II	11	67007621	KDM2A	Body				Promoter_Associated	
347	cg24705286	II	16	27215306	KDM8	1stExon	Island			Promoter_Associated	

Table S1: Annotation of CpG sites in KDM gene family

Seq	Probe	Infinium Design Type	CHR	Position	Gene Symbol	Domain	Relation_to CpG_Island	DMR	Enhancer	Feature_Group	DHS
348	cg24749656	II	2	86668528	KDM3A	5'UTR	Island			Promoter_Associated	TRUE
349	cg24776350	I	17	7757236	KDM6B	3'UTR	Island			Unclassified_Cell_type_specific	
350	cg24823679	II	19	5093491	KDM4B	Body	Island				
351	cg24880665	II	1	202772164	KDM5B	Body	N_Shelf				
352	cg24893073	II	17	7742126	KDM6B	TSS1500	N_Shelf			Unclassified_Cell_type_specific	
353	cg24987648	I	12	498827	KDM5A	TSS1500	Island			Promoter_Associated	
354	cg25014247	II	12	122015908	KDM2B	Body	N_Shore	RDMR			
355	cg25246084	II	19	4971487	KDM4B	5'UTR	S_Shelf		TRUE	Promoter_Associated	TRUE
356	cg25292628	II	9	6758853	KDM4C	5'UTR	Island				
357	cg25312876	I	19	5151581	KDM4B	3'UTR	Island			Unclassified_Cell_type_specific	
358	cg25401482	II	19	5148023	KDM4B	Body	Island			Unclassified_Cell_type_specific	TRUE
359	cg25482146	I	12	122016415	KDM2B	Body	Island				TRUE
360	cg25569575	I	11	67007811	KDM2A	Body				Promoter_Associated	
361	cg25588826	II	17	7755583	KDM6B	Body	Island		TRUE	Promoter_Associated	
362	cg25597833	II	11	66888499	KDM2A	5'UTR	S_Shore			Promoter_Associated_Cell_type_specific	
363	cg25716786	II	12	498630	KDM5A	TSS200	Island			Promoter_Associated	
364	cg25732961	I	17	7755877	KDM6B	Body	N_Shore		TRUE	Promoter_Associated	
365	cg25841161	II	1	44115617	KDM4A	TSS200	Island			Promoter_Associated	
366	cg25949093	I	19	5142198	KDM4B	Body	Island			Unclassified_Cell_type_specific	
367	cg26218726	I	12	122018391	KDM2B	TSS200	Island				TRUE
368	cg26314534	I	17	7757210	KDM6B	3'UTR	Island			Unclassified_Cell_type_specific	
369	cg26345259	II	19	5010347	KDM4B	5'UTR					
370	cg26509318	II	12	122019760	KDM2B	TSS1500	S_Shore	RDMR		Promoter_Associated	
371	cg26662347	II	1	23345754	KDM1A	TSS200	N_Shore			Promoter_Associated	
372	cg26759552	II	7	139877471	KDM7A	TSS1500	Island				
373	cg26800802	II	7	139859464	KDM7A	Body			TRUE		
374	cg26967415	II	17	7748407	KDM6B	5'UTR	Island			Promoter_Associated	
375	cg26993500	I	12	121975121	KDM2B	Body	Island			Promoter_Associated	
376	cg26995224	II	12	121974146	KDM2B	Body	N_Shore	RDMR	TRUE	Promoter_Associated	
377	cg27118526	I	16	27215372	KDM8	1stExon	Island			Promoter_Associated	
378	cg27162932	I	12	121962881	KDM2B	Body			TRUE		

Table S1: Annotation of CpG sites in KDM gene family

Seq	Probe	Infinium Design Type	CHR	Position	Gene Symbol	Domain	Relation_to CpG_Island	DMR	Enhancer	Feature_Group	DHS
379	cg27349129	I	19	5080888	KDM4B	Body	S_Shelf			Unclassified_Cell_type_specific	
380	cg27366994	II	19	5145824	KDM4B	Body	Island				
381	cg27394203	I	17	7752261	KDM6B	Body	N_Shelf				
382	cg27461800	I	12	498354	KDM5A	5'UTR	Island			Promoter_Associated	
383	cg27531483	I	1	202776874	KDM5B	Body	Island				TRUE
384	ch.11.1369160F	II	11	66922185	KDM2A	Body					
385	ch.1.1363396R	II	1	44168444	KDM4A	Body					
386	ch.2.1916207F	II	2	86719269	KDM3A	3'UTR					
387	cg00109780	II	9	96337982	PHF2	TSS1500	N_Shore		NA		
388	cg11181577	II	9	96339738	PHF2	Body	Island		NA		
389	cg13442749	II	9	96337681	PHF2	TSS1500	N_Shore		NA		
390	cg13787134	II	9	96362102	PHF2	Body			TRUE		
391	cg14426288	II	9	96440615	PHF2	3'UTR			NA		
392	cg20684399	II	9	96338308	PHF2	TSS1500	Island		NA		
393	ch.9.1355968F	II	9	96414449	PHF2	Body			NA		
