# Supplementary material for: Epigenetic modifications in KDM lysine demethylases associate with survival of early-stage NSCLC
Source: Clin Epigenetics. 2018 Apr 2;10:41. doi: 10.1186/s13148-018-0474-3 (PMC5879927; doi:10.1186/s13148-018-0474-3)
Supplement: Supplementary file 3 — Table S2. Distributions of CpG sites in KDM genes. (PDF 153 kb) [file 13148_2018_474_MOESM3_ESM.pdf]

Table S2: Distributions of CpG sites in KDM genes

Subfamily	Gene symbol[a]	Gene name	Chromosome	N of CpG sites						
				TSS1500	TSS200	3'UTR	5'UTR	1stExon	Body	**Total**
KDM1	KDM1A	lysine demethylase 1A	1p36.12	1	2	0	0	5	1	**9**
	KDM1B	lysine demethylase 1B	6p22.3	4	3	0	1	0	1	**11**
KDM2	KDM2A	lysine demethylase 2A	11q13.1	5	0	1	3	4	18	**31**
	KDM2B	lysine demethylase 2B	12q24.31	3	11	1	1	1	55	**72**
KDM3	KDM3A	lysine demethylase 3A	2p11.2	6	0	1	10	0	2	**18**
	KDM3B	lysine demethylase 3B	5q31	3	3	1	0	2	2	**11**
KDM4	KDM4A	lysine demethylase 4A	1p34.1	3	5	1	4	3	5	**21**
	KDM4B	lysine demethylase 4B	19p13.3	2	1	2	11	0	78	**94**
	KDM4C	lysine demethylase 4C	9p24-p23	2	0	0	9	0	4	**15**
	KDM4D	lysine demethylase 4D	11q21	0	4	1	2	4	0	**11**
	KDM4E	lysine demethylase 4E	11q21	1	1	1	0	0	0	**3**
KDM5	KDM5A	lysine demethylase 5A	12p13.33	4	1	0	6	3	0	**14**
	KDM5B	lysine demethylase 5B	1q32.1	1	2	0	0	2	6	**11**
	KDM5C[b]	lysine demethylase 5C	Xp11.22-p11.21	–	–	–	–	–	–	–
	KDM5D[b]	lysine demethylase 5D	Yq11	–	–	–	–	–	–	–
KDM6	KDM6A[b]	lysine demethylase 6A	Xp11.2	–	–	–	–	–	–	–
	KDM6B	lysine demethylase 6B	17p13.1	3	0	10	12	0	10	**35**
KDM7	KDM7A	lysine demethylase 7A	7q34	6	0	0	0	0	6	**12**
-	PHF8[b]	PHD finger protein 8	Xp11.22	–	–	–	–	–	–	–
-	PHF2	PHD finger protein 2	9q22	3	0	1	0	0	3	**7**
-	KDM8	lysine demethylase 8	16p12.1	6	0	1	4	3	4	**18**
		**Total**		**53**	**33**	**21**	**63**	**27**	**196**	**393**

TSS, transcription start site; UTR, untranslation region.

[a]The 21 genes are classified into KDM family (http://www.genenames.org/cgi-bin/genefamilies/set/485)

[b]not included in the initial analysis due to its location in sex chromosomes
