# Supplementary material for: Epigenetic modifications in KDM lysine demethylases associate with survival of early-stage NSCLC
Source: Clin Epigenetics. 2018 Apr 2;10:41. doi: 10.1186/s13148-018-0474-3 (PMC5879927; doi:10.1186/s13148-018-0474-3)

- **Figure S3. DNA methylation probes identified by flexible criterion in SCC and their correlation with corresponding gene expression.**
- Three more sites were identified in SCC samples using more flexible criterion: cg06646494 in *KDM2B*, cg00121158 in *KDM4C*, cg06615743 in *KDM4B*. A-B shows cg00121158 and cg06615743 and their correlation with corresponding gene expression.

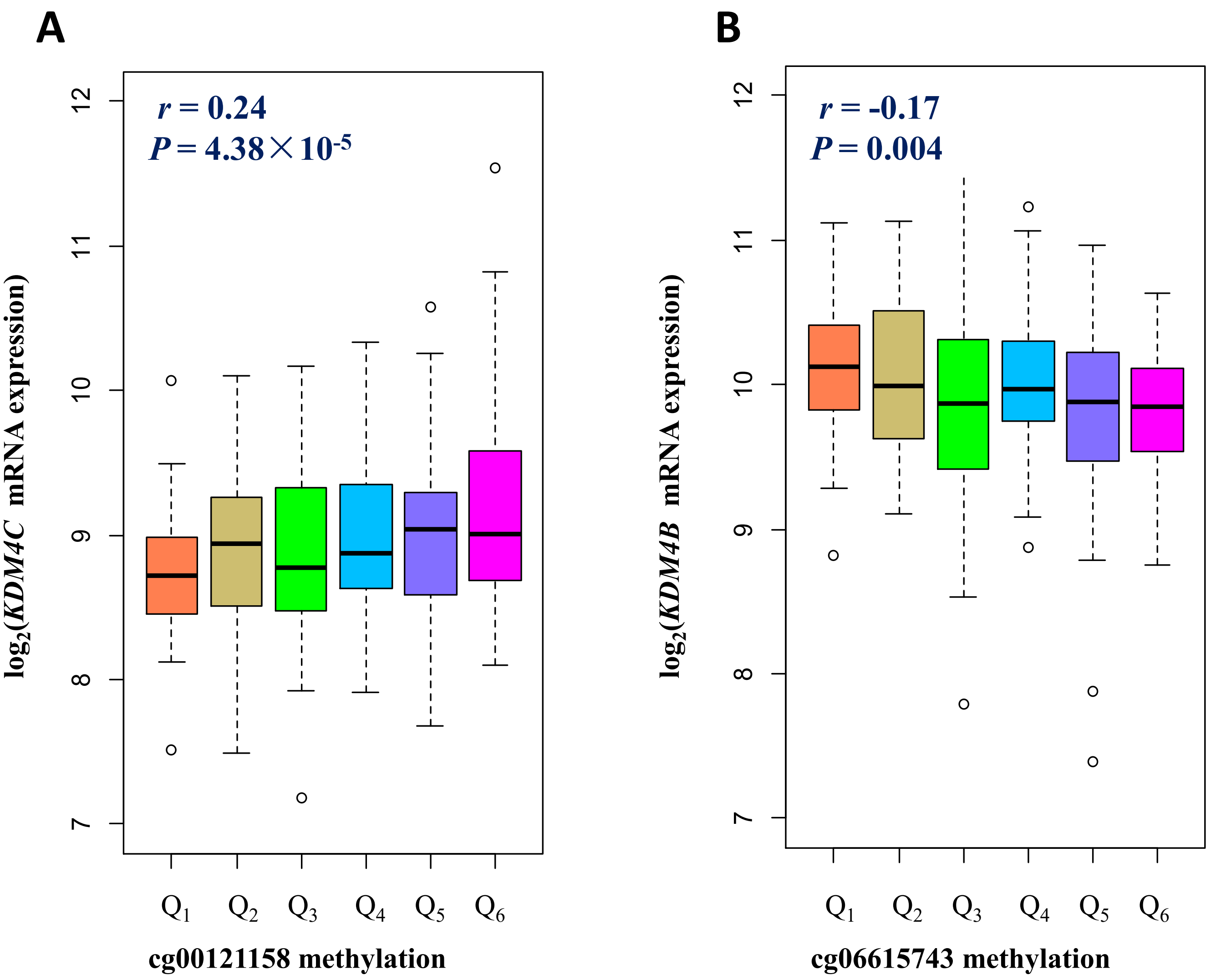

Supplement: Supplementary file 5 — Figure S3. DNA methylation probes identified by flexible criterion in squamous cell carcinoma and their correlation with corresponding gene expression. Three more sites were identified in SCC samples under flexible criterion: cg06646494 in KDM2B, cg00121158 in KDM4C, and cg06615743 in KDM4B. The cg00121158 (A) and cg06615743 (B) showed a statistical correlation with the corresponding gene expression. DNA methylation level was categorized to six quantiles and box plot for gene expression was drawn for each quantile. Pearson correlation was used to estimate the correlation coefficient (r) and the P value, Gene expression was log2 transformed before analysis. (PDF 571 kb) [file 13148_2018_474_MOESM5_ESM.pdf]
